# CD8^+^ T Cells in Chronic Periodontitis: Roles and Rules

**DOI:** 10.3389/fimmu.2017.00145

**Published:** 2017-02-21

**Authors:** Elsa M. Cardoso, Fernando A. Arosa

**Affiliations:** ^1^Health Sciences Research Centre (CICS-UBI), Faculty of Health Sciences (FCS-UBI), Universidade da Beira Interior, Covilhã, Portugal

**Keywords:** CD8^+^ T cells, suppressor, regulatory, cytokines, gingiva, periodontal disease, alveolar bone, tissue repair

## Introduction

Chronic periodontitis is a multifactorial disease characterized by the presence of dysbiotic microbial communities that, together with genetic and environmental factors, results in chronic inflammation of the periodontium, which ultimately may trigger alveolar bone resorption (1). The reported associations between periodontal disease and other chronic disorders, including metabolic, cardiovascular, respiratory, and arthritic diseases (2), reinforce the importance of elucidating the cell types and molecular mechanisms involved. Chronic inflammation of gingival tissue has long been associated with infiltration of the gingiva by activated T and B cells and secretion of inflammatory cytokines and immunoglobulins. However, the role of T cells in periodontal disease is controversial with reports showing that they have both protective and destructive roles (3, 4). Moreover, while most studies have focused on CD4^+^ T cells and B cells, the role played by gingival CD8^+^ T cells has been overlooked. On the basis of data published recently, we discuss the possibility that gingival CD8^+^ T cells contain a pool of cells with regulatory/suppressor properties involved in the maintenance of gingival tissue integrity by constitutively downregulating inflammation under homeostatic conditions and initiating repairing mechanisms in case of tissue injury. These basic physiological roles could be surpassed and hidden when a potent and/or chronic immune response against pathogenic bacterial colonization occurs, thus leading to bone loss. Elucidation of the basic physiological roles of particular CD8^+^ T cells present in periodontal tissue and the rules they follow in order to cope with minor versus major disruption of tissue homeostasis can improve our understanding of how they react to changes in their environment and ultimately allow the development of novel therapeutic approaches to favor anti-inflammatory responses and bone repair.

## The Roles of CD8^+^ T Cells: Cytotoxicity, Suppression, and Tissue Repair

One fascinating aspect of CD8^+^ T cells is their heterogeneity, as they differ in terms of T cell receptor (TCR) diversity and antigen specificity, which allows them to monitor for shifts in peptide antigens presented in MHC class I molecules expressed on the plasma membrane of all nucleated cells. CD8^+^ T cells acquire functional properties after being activated, normally in secondary lymphoid organs, by antigen presenting cells (APC). As a result, some acquire innate receptors, including NK receptors, enlarging the kind of stimuli they can receive (5). In addition to the well-documented cytotoxic activities, CD8^+^ T cells might also have regulatory/suppressor functions (thereafter CD8^+^ Treg) since they have the ability to control other leukocytes to avoid excessive immune activation and its pathological consequences (6, 7). Although in humans many phenotypes have been described for CD8^+^ Treg, the most reliable maker is the transcription factor Foxp3 (7). Nevertheless, CD8^+^ Tregs have also been described as Foxp3^low^ (8) and Foxp3^−^, including CD8^low^CD28^−^, CD8^+^CD103^+^, and non-antigen-specific CD8^+^CD28^−^ (9–11). CD8^+^ Tregs are also diverse regarding the mechanisms of suppression, which include induction of tolerogenic APC, withdrawal of homeostatic cytokines, secretion of anti-inflammatory cytokines, and cell cytotoxicity (6, 7). The importance of CD8^+^ Treg in the context of tissue inflammation is illustrated by a series of studies showing that interactions between intestinal epithelial cells and CD8^+^ T cells induce a population of CD8^+^CD28^−^CD103^+^ T cells endowed with suppressor functions (12, 13). Subsequent studies by the same group demonstrated that the disruption of their suppressor activities is associated with mucosal inflammation (8). The regulatory functions of highly differentiated CD8^+^ T cells might also include tissue repair (14), although it has been mainly described in CD4^+^ Treg for lung (15), and αβ and γδ T cells for bone (16, 17). Indeed, the experimental evidence for the existence of CD8^+^ T cells endowed with tissue repair and/or bone regeneration properties within the gingival tissue is very scarce as described below, which warrants further studies.

## CD8^+^ T Cells and Bone Homeostasis: Cytokines Matter

An early seminal study using NOD/SCID mice transplanted with human peripheral blood lymphocytes as a model of periodontitis showed that human CD4^+^ T cells in the periodontium triggered local alveolar bone destruction by secreting osteoprotegerin ligand, also known as RANKL (18). Subsequently, it was demonstrated that several pro-inflammatory cytokines, for example TNF-α, ultimately converge on the expression of RANKL thus promoting osteoclastogenesis (19). In contrast, anti-inflammatory cytokines secreted by CD8^+^ T cell with an effector–memory phenotype, such as IL-10 and TGF-β (7, 20), have been shown to be bone protective in *in vivo* and *in vitro* models of bone regeneration (19, 21, 22). The role of pro-inflammatory cytokines IL-17 and IFN-γ in bone homeostasis remains controversial. IL-17 is mainly produced by CD4^+^ Th17 cells and γδ T cells and has been associated with bone destruction (23). However, recent experimental studies in knockout mice models have shown that IL-17 may participate in the early phases of bone regeneration by directly stimulating osteoblastogenesis (16, 17). Regarding IFN-γ, it has been shown to promote as well as to inhibit bone formation (23–25). The contrasting effects of IFN-γ could perhaps be explained by the fact that it may exert a direct inhibitory effect on osteoclastogenesis by interfering with the RANK pathway, and at the same time promote bone destruction indirectly by inducing antigen-presenting MHC molecules on APC, leading to increased production of TNF-α by activated T cells (26). Whether the IFN-γ discrepancies on bone homeostasis may result from the study of early versus late phases of bone formation, as it happens with IL-17, remain to be elucidated. Importantly, recent studies in models of bone regeneration have shown that mouse CD8^+^ T cells and *in vitro* expanded human CD8^+^ T cells secrete Wnt10b, a cytokine/factor that promotes osteoblastogenesis (27). Even though the exact mechanism used by CD8^+^ T cells to promote bone regeneration remains to be elucidated, the accumulated evidence from experimental models of bone regeneration suggests that cytokines and factors secreted by CD8^+^ T cells could be involved.

## Gingival CD8^+^ T Cells and Chronic Periodontitis

The majority of studies on gingival tissue of chronic periodontitis focused on the functional characterization of CD4^+^ T cells and B cells and concluded that the presence of CD4^+^ Th1 cells and antibody-secreting B cells, as a result of the host immune response against bacterial infection, was associated with chronic inflammation and disease progression, namely alveolar bone loss (1). Indeed, a detrimental role of gingival CD4^+^ T cells in alveolar bone destruction under chronic periodontitis has been steadily proposed since the seminal work of Penninger’s group (18). These conclusions are supported by an *in vivo* study in CD4- and CD8-deficient mice showing that CD4^+^ T cells contribute to the alveolar bone loss in mice (28). Although CD4^+^ Treg cells have been shown to confer protection in animal models of periodontal disease (29, 30), they are present in low numbers in gingival tissue of subjects with chronic periodontal disease (31, 32) and some of them may switch to an inflammatory Th17 phenotype (33).

**Figure 1:**
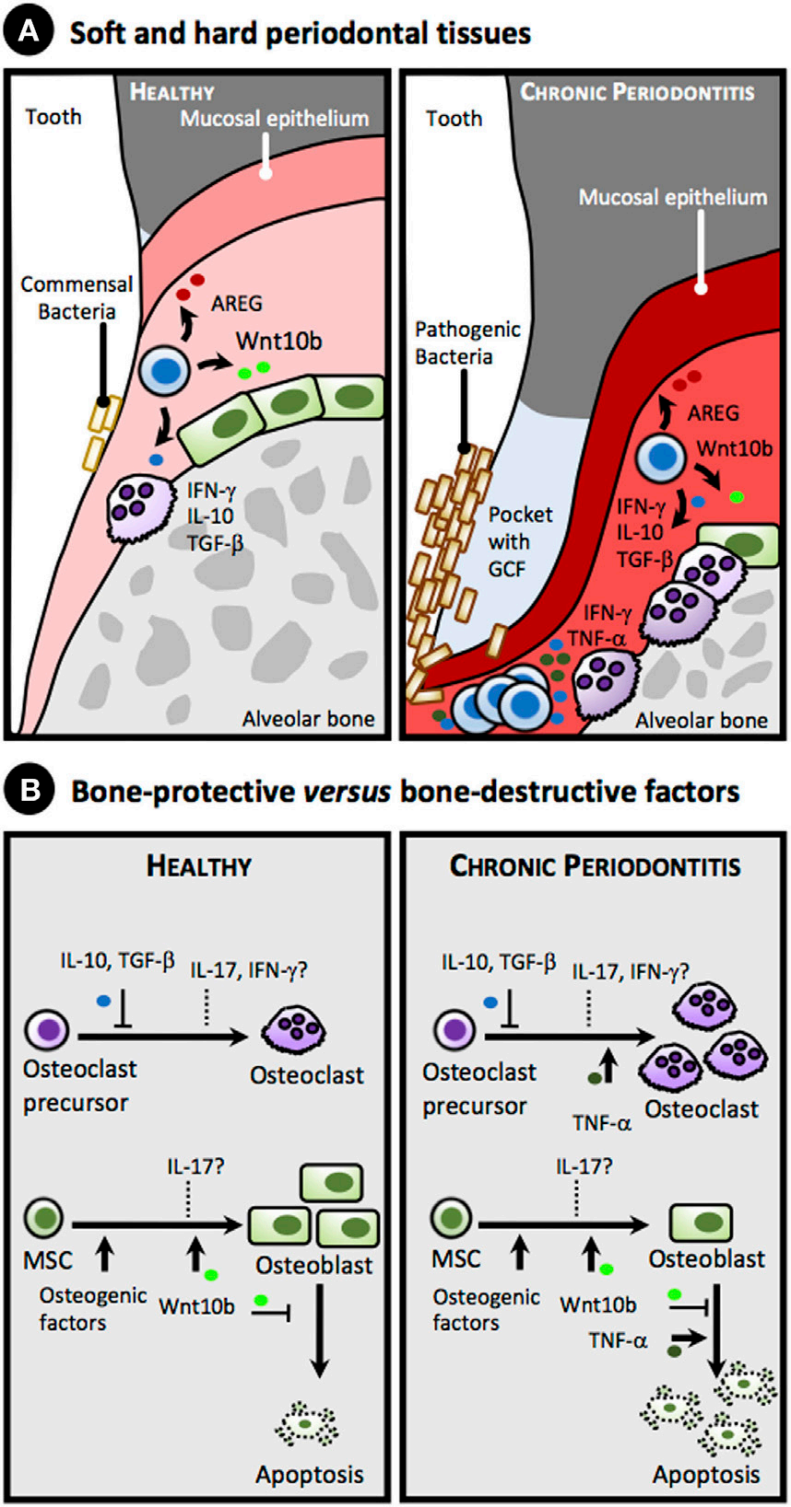
**Proposed simplified model for the role of gingival CD8^+^ T cell-derived cytokines and factors in alveolar bone homeostasis in health and chronic periodontitis**. **(A)** Anatomy of soft and hard periodontal tissues showing selected cells: CD8^+^ T cells, herein collectively referred as memory CD8^+^ T cells or T_M_ (blue), osteoblasts (green), and osteoclasts (purple), in healthy periodontal tissues (left panel) and in chronic periodontitis (right panel). Note accumulation of pathogenic bacteria on the tooth surface, formation of a pocket with gingival crevicular fluid (GCF) accumulation, gingival attachment loss, inflamed gingiva (reddish), as well as loss of alveolar bone in chronic periodontitis due to imbalance in the equilibrium between osteoblastogenesis and osteoclastogenesis toward the latter (right panel). **(B)** Bone-protective versus bone-destructive factors that can be produced by gingival CD8^+^ T_M_ cells. Under homeostatic conditions (left panel), IL-10 and TGF-β secreted by CD8^+^ T_M_ cells suppress osteoclastogenesis, while Wntb10 promotes differentiation of mesenchymal stem cells (MSC) into osteoblasts and inhibits their apoptosis. In addition, CD8^+^ T_M_ cells secrete amphiregulin (AREG) that downplays gingival inflammation and promotes epithelial and stromal tissue repair (data not shown, see text). Overall, these bone promoting cytokines counteract the bone loss induced by commensal bacteria (see text). The role of IFN-γ and IL-17 under homeostatic conditions on osteoclastogenesis and osteoblastogenesis is uncertain (dashed lines). Under chronic periodontitis (right panel) high levels of TNF-α, and perhaps of IFN-γ and IL-17, produced in response to pathogenic bacterial colonization surpass the protective role of the aforementioned cytokines, promoting osteoclastogenesis and increasing apoptosis of osteoblasts, thus favoring bone loss.

Although the role of CD8^+^ T cells in chronic periodontitis is less obvious, most studies have consistently shown that despite being more abundant in gingival tissues of periodontitis patients than in patients with gingivitis or healthy controls, CD8^+^ T cells are not involved in gingival tissue pathology (3, 4). Similar conclusions were drawn from mice studies (28). Interestingly, a recent comprehensive study using multiparametric flow cytometry has revealed that T cells present in healthy gingival tissue are predominantly effector–memory, as determined by the use of CD45RA, CD45RO, and CCR7, with CD4^+^ and CD8^+^ T cells being more abundant than B cells (34). In gingival tissue from chronic periodontitis, a marked increase in total CD4^+^ T cells, CD8^+^ T cells, and B cells, akin to neutrophils, was observed, and most of the CD4^+^ T cells produced IL-17 (34). Although in the study of Dutzan et al. CD8^+^ Treg are not detected in the gingiva of chronic periodontitis patients (34), previous studies showed that some gingival CD8^+^ T cells lack CD28 while expressing the inhibitory receptor PD1 (35–37). These features are associated with an effector–memory phenotype (20). These data, together with early *in vitro* studies showing that CD8^+^ T cells confer bone protection by suppressing osteoclastogenesis (38, 39), suggest that effector–memory CD8^+^ T cells present in gingival tissue might play a key basic physiological role in safeguarding tissue integrity (Figure 1). Nevertheless, further studies are needed to ascribe a particular protective role to specific CD8^+^ T cells. Indeed, this role may be overwhelmed and masked by inflated T and B cell immune responses against bacterial aggression under dysbiotic bacterial growth, with dramatic outcomes for bone homeostasis.

## Gingival CD8^+^ T Cells: Molecular Signals and Effector Functions

Given the effector–memory phenotype (34–37), and unlike CD4^+^ T cells that express CD28 and may respond to TCR/CD28-mediated signals, gingival CD8^+^ T cells may preferentially be activated in a TCR-independent manner by local signals produced during stress and/or injury, including a variety of endogenous products that signal through innate receptors, as discussed elsewhere (20). As a result, upon innate receptor triggering gingival CD8^+^ T cells may secrete cytokines, such as IL-10 and IFN-γ, reported to have bone repairing properties (23, 26). Interestingly, a recent study showed that, unlike inflammatory cytokines, the levels of IL-10 remained unchanged in the gingival crevicular fluid of chronic periodontitis patients after non-surgical periodontal therapy (40), suggesting that IL-10 may have indeed a basic physiologic role in the healthy gingiva, as demonstrated in IL-10-deficient mice (41). In addition, gingival CD8^+^ T cells could further improve tissue healing after receiving environmental signals by secreting amphiregulin, an anti-inflammatory cytokine expressed by CD8**^+^** T cells (42), which has been shown to promote tissue repair (14), and is upregulated in the gingival stroma in a mice model of chronic periodontitis (43) (Figure 1).

Evidence for the expression of innate/inhibitory receptors by gingival CD8^+^ T cells, including KIR, LIR, TLR, and others, is very scarce, which warrants the need and importance of studying their expression. Thus, though initially considered a T cell exhaustion marker, the reported expression of PD1 by gingival CD8^+^ T cells (37) could potentially be involved in limiting tissue damage through interaction with its ligand, which can be expressed by a variety of stromal cells (44, 45). On the other hand, *in vitro* studies in mice have recently proposed that CD8^+^ T cells could be activated by osteoclasts *via* antigen cross-presentation, resulting in the formation of CD8^+^ Treg that could inhibit bone resorption through secretion of IFN-γ (46). These results are challenging and suggest that bone-protective CD8^+^ T cells could be generated *in loco* from resident CD8^+^ T cells in the alveolar bone surface, while bone-protective CD4^+^ Treg may be recruited from circulation (29, 30). In this respect, it is important to mention that the commensal bacteria present in the gingiva may exert an important role in alveolar bone homeostasis. Thus, a series of animal studies performed in germ-free *versus* specific pathogen-free *versus* wild-type models have shown that commensal bacteria present in the gingiva is responsible for physiological alveolar bone loss (1, 47), suggesting that gingival T cells and their secreted cytokines might be present from birth, as seen in other peripheral tissues (48), and contribute to physiologic alveolar bone homeostasis in healthy conditions.

## Concluding Remarks and Future Prospects

While scant, there is evidence that resident gingival CD8^+^ T cells may contain lymphocytes with regulatory functions, including suppression of bone-destructive cytokines and repair of alveolar bone, two activities that could be intertwined. However, the host immune response that takes place upon chronic bacterial colonization of the teeth and that results in the recruitment of innate and adaptive inflammatory cells (34) will likely disrupt these homeostatic activities. Thus, it turns out of the foremost importance to elucidate the role of gingival effector–memory CD8^+^ T cells in bone remodeling in health and sickly conditions. To do that, the use of mice models lacking selected populations (cytotoxic CD8^+^ T cells versus CD8^+^ Treg, etc.), suppressive cytokines or tissue-specific chemokine receptors, or adoptive transfer of CD8^+^ Treg should provide insights into the bone-protective role of gingival CD8^+^ T cells (30, 49, 50). These studies will certainly broaden our understanding of the relationship of gingival CD8^+^ T cells with the periodontium and enable the development of novel therapies to inhibit bone loss under pathological conditions.

## Author Contributions

EC did bibliography search and wrote the manuscript. FA did bibliography search and edited the manuscript.

## Conflict of Interest Statement

The authors declare that the research was conducted in the absence of any commercial or financial relationships that could be construed as a potential conflict of interest.
